# Evaluation of a Proposed Approach for the Determination of the Bioequivalence Acceptance Range for Narrow Therapeutic Index Drugs in the European Union

**DOI:** 10.3390/pharmaceutics14112349

**Published:** 2022-10-31

**Authors:** Paulo Paixão, Nuno Silva, Rita Bento Guerreiro, Kevin Blake, Milton Bonelli, José Augusto Guimarães Morais, Alfredo García-Arieta, Luís Filipe Gouveia

**Affiliations:** 1 Research Institute for Medicines (iMed.ULisboa), Faculty of Pharmacy, Universidade de Lisboa, 1649-004 Lisboa, Portugal; 2 EMA’s Pharmacokinetics Working Party, 1083 HS Amsterdam, The Netherlands; 3 European Medicines Agency (EMA), 1083 HS Amsterdam, The Netherlands; 4 The Spanish Agency of Medicines and Medical Devices, 28022 Madrid, Spain

**Keywords:** narrow therapeutic index, bioequivalence, medicines regulation, generic medicinal products

## Abstract

Bioequivalence (BE) of products containing narrow therapeutic index (NTI) drugs in the European Union is currently established by demonstrating that the 90% confidence interval for the ratio of the population geometric means of the test compared to the reference product’s AUC, and in certain cases Cmax, is included within the tighter acceptance range of 90.00–111.11%. An alternative criterion, consisting of narrowed limits based on the within-subject variability of the reference product, was recently proposed. Its performance for a three-period partial replicate design was tested by simulation in terms of power to show BE, type I error (T1E) and sample size requirements. A new condition, a constraint on the test-to-reference geometric mean ratio (cGMR) to be contained within the range of 90.00–111.11%, was also tested. The probability of showing BE when the products differ more than 10% was increased, but only if the reference product’s within-subject variability was moderate-to-high. The inclusion of the additional cGMR limited this. An increase in the T1E (<7%) was observed. The inclusion of the additional cGMR did not change the highest inflation of the T1E. Finally, a significant sample size reduction was observed and the inclusion of the cGMR usually did not increase the required sample size.

## 1. Introduction

Bioequivalence (BE) between two drug products results in the conclusion that both, by showing a sufficiently similar rate and extent of absorption, should present a comparable in vivo performance in terms of safety and efficacy. In general, BE studies are usually conducted in healthy subjects, where the plasma concentration—time curve is generally used to assess the rate and extent of absorption [1]. The assessment of BE is based upon the 90% confidence interval (CI) for the test-to-reference product population geometric mean ratio (GMR) for the parameters under consideration, usually area under the concentration time curve (AUC), that reflects the extent of exposure, and the maximum plasma concentration (Cmax), or peak exposure, that is influenced by the absorption rate, using an average bioequivalence (ABE) approach [2].

BE is usually concluded if the GMR 90% CI falls within the regulatory acceptance limits. Usually, it is considered that a ±20% difference between the test and reference Cmax and AUC should not result in clinically relevant differences and based on this, these limits are fixed and symmetrical on a logarithmic scale presenting an acceptance range on the original scale of 80.00–125.00% [3]. However, for a narrow therapeutic index (NTI) drug, where a small difference in the administered dose may result in either serious therapeutic failures or adverse drug reactions [4], a more conservative approach is usually followed by considering an acceptable difference reduced to ±10% [5]. In these situations, a tighter acceptance interval of 90.00–111.11% (thereafter referred to as ‘tighter limits’) is currently applied arbitrarily by the European Medicines Agency (EMA) [6], as well as several other regulatory agencies [7,8], resulting in an increased difficulty to demonstrate BE because of larger sample size requirements. It is considered unnecessary to narrow the acceptance range by more than this ±10% acceptance range because the reference product content can vary between 95% and 105% in the European Union, therefore, the reference side batches may differ up to 10%, even if it is acknowledged that the difference in content and the differences in bioavailability are additive factors.

Recently, an alternative acceptance criterion has been proposed for products containing NTI drugs that would allow for a lower burden in terms of the number of subjects required to show BE [9]. This approach, that intents to be used as an alternative option on a voluntary basis to the current European NTI acceptance criteria, consists of an ABE with narrowed limits based on the intra-subject (or within-subject) variability of the reference product (NLIVR), similarly to the approach used for widening the acceptance range of Cmax in the case of highly variable drug products (HVDP) [6]. If the applicant decides to take use of this approach, the following five conditions have been proposed:

(1) The within-subject standard deviation (s_WR_) is calculated from the reference formulation in the same replicate cross-over study where the acceptance range is to be narrowed;
(2) If the estimated reference within-subject coefficient of variation (WSCV) does not exceed 13.93% (corresponding to s_WR_ ≤ 0.1386), the 90.00–111.11% acceptance range is applied;
(3) If the estimated WSCV exceeds 30% (corresponding to s_WR_ ≥ 0.29356), the 80.00–125.00% acceptance range is applied;
(4) If the estimated WSCV range between 13.93% and 30%, the acceptance range is defined by [U, L] = exp [±k·s_WR_];
(5) The regulatory “proportionality” constant k is set to 0.760, as for HVD products.

**Figure 1:**
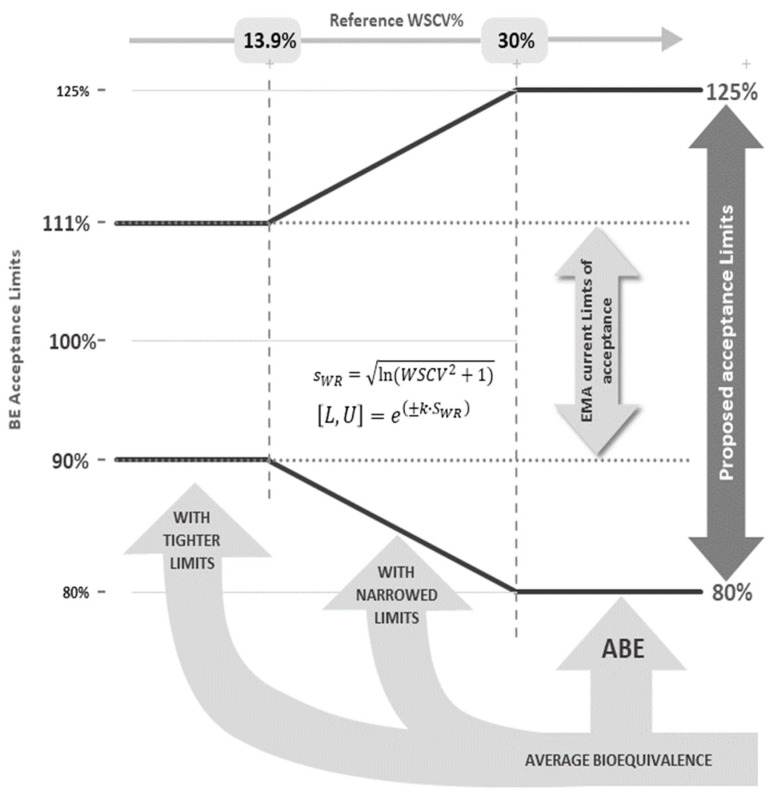
Acceptance limits for the 90% CI of the test-to-reference GMR of NTI drugs according to the WSCV of the reference product.

A graphical representation of the proposed strategy is shown in Figure 1. In theory, and because the acceptable differences between test and reference are similar to the differences that may exist within the reference product itself, this approach is not expected to increase the clinical risk. In fact, in the past some NTI drugs were approved with a 20% acceptance range (e.g., carbamazepine, levothyroxine) in the European Union and presently some drugs considered as NTI drugs in the US FDA are not considered as NTI drugs in the European Union (e.g., dabigatran, flecainide). Additionally, the advantages of the method in terms of reducing the number of subjects have already been previously discussed [9]. However, similarly to what was noted for the HVDP ABE with expanding limit criteria [10], the TOST (two one-sided test) procedure cannot be directly applied to the proposed NLIVR method since the BE limits themselves become random variables and the method is not correct in the strict sense. As such, in this work, the authors intend to explore the performance of the procedures to be applied for the determination of BE for products containing NTI drugs using the NLIVR criteria, by means of power curves that were simulated under various assumptions, conditions and sample size requirements. Since, theoretically, the NLIVR criteria will allow for products with higher differences to be considered as bioequivalent, the application of a new and additional requirement on the method (a further condition 6); namely, a constraint on the GMR to be contained in the acceptance range of 90.00–111.11%, was also tested.

## 2. Materials and Methods

R version 3.6.2 (Platform x86_64_264-mingw32/x64, 2019, The R Foundation for Statistical Computing) [11] was used for simulations on a PC with an Intel Core i5-4200U processor and 4Gb RAM. The package PowerTOST [12] for the statistical language R was used for all calculations. 

### 2.1. Power Analysis

For defining the overall power curves for a two-treatment, three-sequence (TRR-RTR-RRT), three-period (2 × 3 × 3) partial replicate design, the number of subjects in the simulations were varied from 9 to 114 (in steps of 3 subjects) and the within-subject variability of the reference product was varied from 5% to 40% (in steps of 0.125%). The function power.scABEL, including a user-defined reg_const additional function, for characterizing the proposed NTI criterium on the PowerTOST package was used. One million BE studies were simulated for each of these individual number of subject/within-subject reference variability conditions, assuming a GMR of 0.9, 0.875, 0.85, 0.825 and 0.8. The final power results represented the percentage of studies concluding BE in each simulated scenario.

### 2.2. Type I Error

For the estimation of the type I error (consumer’s risk—T1E) a similar protocol to the power analysis was performed. However, the GMR values were varied depending on the within-subject coefficient of variation of the reference formulation according to:

GMR = 0.90 if WSCV ≤ 13.92%

GMR = e^−0.76 σ^^WR^ if 13.92% < WSCV < 30.00%

GMR = 0.80 if WSCV ≥ 30.00%

i.e., just over the lower limit of the acceptance range and under homoscedasticity (WSCV of test = WSCV of reference). Ideally, in these cases, the type I error should be exactly equal to the significance level 𝛼 [13]. Based on the binomial test with this number of simulations an empirical T1E rate above 0.05036 was shown to be considered statistically significantly inflated [14].

### 2.3. Sample Size

The sample size is calculated via iterative evaluation of power of the TOST procedure calculated as the success rate of showing BE as the sample size increases. For each BE metric (AUC and Cmax), in order to calculate the sample size for a BE trial, defining the following is needed (a) the one-sided significance level α, with a value of 0.05 (as commonly accepted by regulators), (b) the type-II error β (which defines the power of the trial (1-β)) (c) the BE acceptance range, (d) the expected GMR for the BE metrics, and (e) the WSCV. In the current EMA criterion, the acceptance range for NTI drugs is defined by the tighter 90.00 to 111.11% limits. For the proposed approach, the BE acceptance range is defined as shown in Figure 1, considering the changes in the criteria depending on the WSCV of the reference drug product. In this work, the GMR was varied from the scenario of no difference between products, with an expected GMR for the BE metrics of 1, to an increasing expected difference of 2.5%, 5% and 7.5% (corresponding to a GMR of 0.975, 0.95 and 0.925, respectively) and a power of 80% or 90%. The WSCV was varied from 5% to 40% (in steps of 0.125%). The same within-subject variability was assumed for the test and the reference products: however, when needed, σ_WR_ was estimated only from the data of the reference product. The study design for all scenarios was the two-treatment, three-sequence (TRR-RTR-RRT), three-period (2 × 3 × 3) partial replicate design. For the current EMA NTI BE criterion, the sample sizes were calculated based on the “Exact” method on the sampleN.TOST function of the PowerTOST package, on which calculations are performed based on formulas with Owen’s Q method [15]. For the new proposed criterion, the sample sizes were predicted based on simulations performed with the sampleN.scABEL function considering again the user define reg_const function on the PowerTOST package [12]. Each calculated sample size represents the minimum number of subjects that provides the target power, assuming a balanced number of subjects per sequence. In other words, the results were rounded upwards, and at the given sample size, the power was at least as high as the stated level.

## 3. Results

### 3.1. Power Analysis

**Figure 2:**
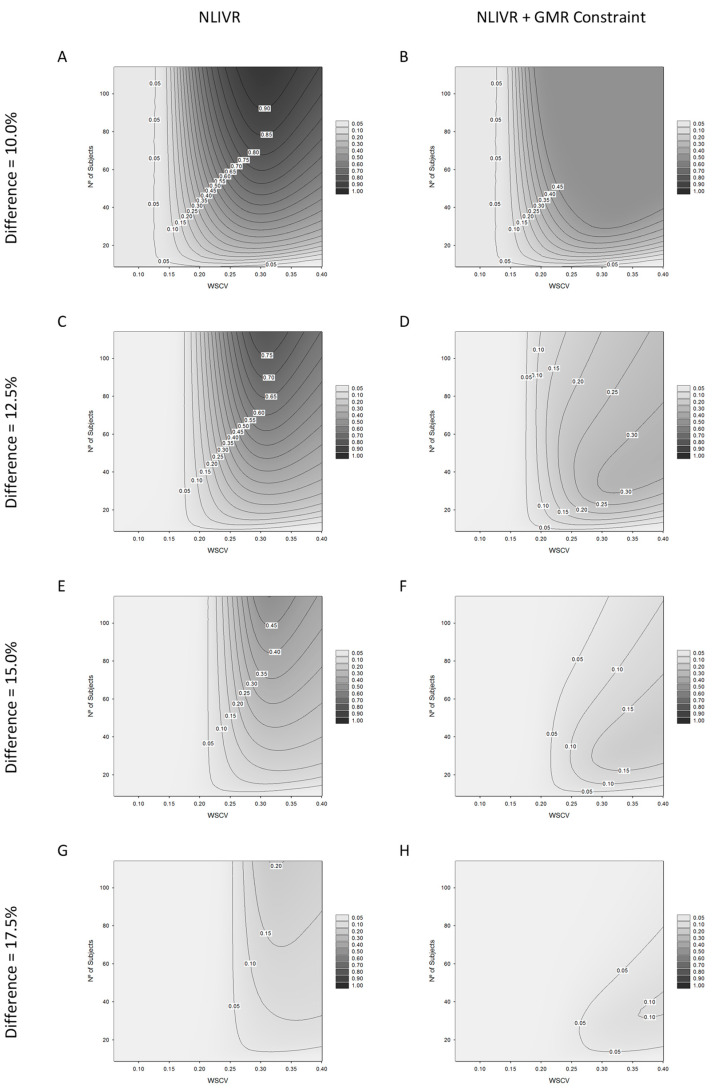
Power analysis for the proposed NLIVR conditions with and without a GMR constraint assuming increasing nominal differences between the test and reference formulations, for different WSCV and number of subjects. Legend represents the probability of concluding BE. (**A**,**C**,**E**,**G**) are related to the NLIVR conditions and (**B**,**D**,**F**,**H**) for the NLIVR with a GMR constraint, for each of the corresponding nominal differences from 10% to 17.5%.

The results from the power analysis can be seen in Figure 2. The left plots are the power curves relationship between the within-subject variability of the reference product and the number of subjects included in the simulated BE trial for the proposed NLIVR criterion. The right plots are the same relationship for the NLIVR criterion with the additional GMR inside the 90.00–111.11% constraint. Overall, these plots show the probability of a test product, with a particular difference from the reference product, to be concluded as BE for a defined number of subjects and within-subject variability of the reference product. It can be seen, from the shift of the 0.05 curves to the right of the plots as the difference between formulations increase, that the probability of concluding BE is always lower than 5% when the difference between the formulations is higher than the WSCV in all the studied scenarios, as expected by the use of a conservative regulatory “proportionality” constant k, that was set to 0.760.

In addition, with the increase in WSCV and the number of subjects, there is a higher probability to show BE between products that can be as high as 90% for products differing by 10% in the studied scenarios (Figure 2A). This power, however, decreases as the theoretical difference approaches the 20% value, where the probability is always less than 5% (plot not shown). The inclusion of the additional GMR constraint inside the 90.00–111.11% range significantly changes the power surface in all situations. The highest probability of concluding BE can only be around 50% if the theoretical difference between the two formulations is larger than 10% (Figure 2B). It is also observed that, in these conditions and as the theoretical difference between the formulations increases, increasing the number of subjects in the study can result in a loss of the general ability to conclude for BE (Figure 2D,F,H).

### 3.2. Type I Error

**Figure 3:**
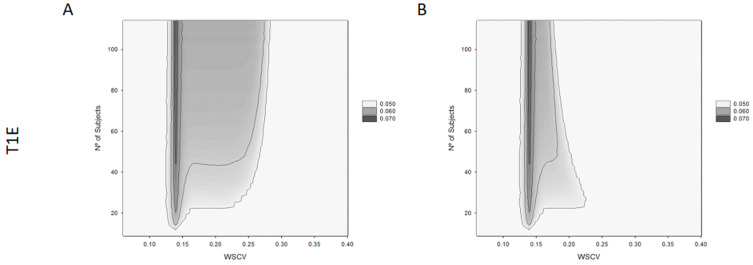
Type I error (T1E) for the proposed NLIVR conditions with (**B**) and without (**A**) GMR constraint for different WSCV and number of subjects.

The results from the T1E analysis can be seen in Figure 3. The left plot (Figure 3A) shows the ability to conclude BE at the lower limit of the acceptance range, in relation to a homoscedasticity WSCV and sample size, for the proposed NLIVR criterion. The right plot (Figure 3B) shows the same relationship for the NLIVR criterion with the additional GMR inside the 90.00–111.11% constraint. In both situations, there is a slight inflation in the T1E mainly at around the WSCV of 13.93%, the cut-off value for the variability where the BE limit for unscaled average BE would start to be expanded proportional to the variability. This value is always below 7%. Inclusion of the GMR constraint does not change the highest inflation in the T1E, which is in the same “switching” zone, but the GMR constraint clearly reduces the overall area of T1E inflation.

### 3.3. Sample Size

**Figure 4:**
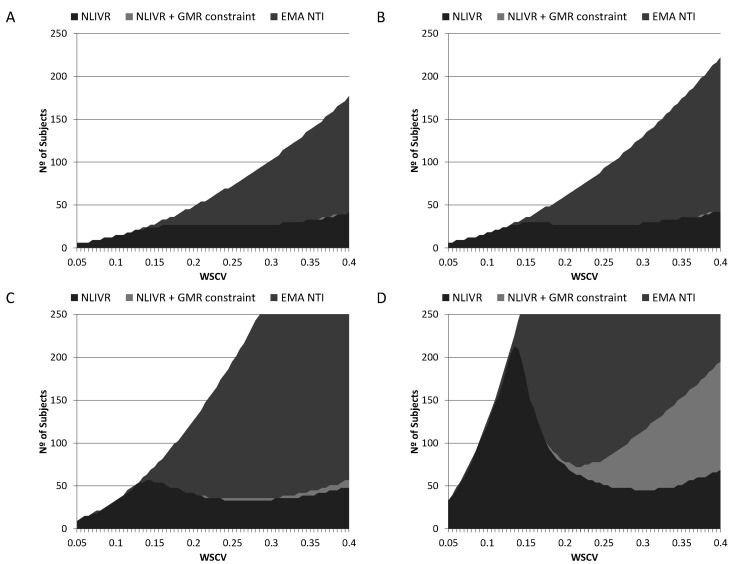
Sample sizes for the EMA current NTI criteria (EMA NTI), the proposed NLIVR conditions with (NLIVR + GMR constraint) and without (NLIVR) the GMR constraint for a power of 80%, assuming a GMR of (**A**) 1.000, (**B**) 0.975, (**C**) 0.950 and (**D**) 0.925.

Sample size requirements to conclude BE with a power of 80% for different nominal differences between products can be seen in Figure 4 and Table 1 for the current EMA NTID fixed 90.00–111.11% acceptance interval, the proposed NLIVR criterion and the NLIVR criterion with the additional GMR inside the 90.00–111.11% constraint. Sample size requirements to conclude BE with a power of 90% in the same conditions can also be seen in Table 1. If the two formulations are equal (GMR = 1), the inclusion of the GMR constraint has no effect on the sample size requirements of the NLIVR assessment, which are increasingly lower than the current EMA requirements for WSCV > 14%. However, small differences start to be seen for WSCV > 25% if a 5% difference (GMR = 0.95) is considered and large sample size differences between the two proposed NLIVR approaches are seen if a difference of 7.5% (GMR = 0.925) is considered. In this case, sample sizes are notably different depending on the WSCV, being as low as 45 subjects for a WSCV of 30% for the NLIVR criterion and 78 subjects for a WSCV of 20% if the GMR constraint is also considered.

## 4. Discussion

We have recently proposed an alternative criterion for assessing BE between two products containing a NTI drug in the European Union, consisting of narrowed limits based on the within-subject variability of the reference product [9], to be used voluntarily by the sponsors if the intra-subject CV is expected to be lower than 13.9%. One of the problems with NTI drugs, especially if they present a low WSCV, is the risk of “generic drift” due to the possibility that an over dimensioned study, with an unnecessarily high number of subjects, could allow to approve a drug product closer to the extremes of the acceptance interval. As such, the approach taken by the EMA (and other regulatory agencies) was to reduce the acceptance difference to 10% and tightened the acceptance interval to 90.00–111.11%. This resulted not only in generic products that are safe and efficacious, but also considered bioequivalence between themselves [5]. However, the definition of NTI drugs in Europe is made on a case-by-case analysis and is based on clinical considerations [6]. Although taken in consideration, having a very low within-subject variability is not a mandatory condition for a drug being regarded as NTI, and examples exist in this regard [9]. As such, for these drug products, higher variability may make it very difficult to conclude BE unless a significantly high number of subjects is recruited. By considering acceptance limits that are narrowed based on the intra-subject CV of the reference product instead of the currently used tighter acceptance interval of 90.00–111.11%, this burden is substantially reduced, but the probability of showing BE when the products differ more than 10%, although expectably clinically irrelevant, is obviously possible if the reference product’s WSCV is moderate-to-high.

The general performance of the proposed regulatory criterion can be seen In Figure 2, either with (right side) or without (left side) the consideration of the additional GMR constraint, where the GMR has to be located inside the 90.00–111.11% acceptance limits. If on the one hand, as theoretically expected, these plots show that the probability to conclude BE between products is always very low when the difference between these drugs is higher than the reference product’s within-subject variability; on the other hand, they also show that, as variability increases, it is possible to approve products with theoretical differences equal to or higher than 10%. For example, if a BE trial with 90 subjects is performed with products differing by 12.5% (FIG 2C), the probability to conclude BE is around 70%, if the reference product presents a within-subject variability higher than 30%. Based on this, it could be argued that a generic product containing a NTI drug could still show BE in spite of its different bioavailability/lower biopharmaceutical quality just by increasing the number of subjects to an acceptable value. However, since the reference product itself shows high variability (>30%) and typically patients are exposed to notable exposure differences between administrations without clinical relevance, this difference > ±10% between the test and reference products should not be clinically relevant. In fact, the differences in bioavailability experienced by the patients when administered chronically is the sum of the differences between units of the same batch, between batches of the same product, the differences due to intra-subject CV and the difference in BA between formulations. The latter is only affected when changing between reference and generic products. For this reason, we may contrast variability of within-patient exposure to a change in the population mean exposure, since both are impacting the exposure differences that are observed in a given patient. If the variability is low, the difference has to be low to consider that the distributions of the test and the reference sufficiently overlap. Consequently, the comparison of differences in ABE can be improved if the differences are assessed under standardization, i.e., when the differences are scaled by the observed variability [16]. However, it could nevertheless result in a general public concern and loss of confidence in generics [17] because a higher theoretical differences between drug products may, in fact, be observed. This same concern was also put forward when the widening of the acceptance range was established for assessing BE of highly variable drug products, fostering the inclusion of an additional point estimate constraint mainly due to “political” reasons [18]. In this line of reasoning, for HVDP under the EMA guideline, the GMR for Cmax must be inside the 80.00–125.00% limit in order to conclude BE within the enlarged acceptance limits [6]. Additionally, although in a different context, under the Canadian guideline, BE for Cmax of “typical” drugs is concluded if the T/R GMR (as point estimate) for this parameter is inside the 80.00–125.00% limits [7].

As shown in Figure 2, the inclusion of this constraint changes the approval surface profile significantly. By being a point estimate-based criterium, this additional condition limits the probability of accepting BE between products differing by 10% at around 50% maximum (Figure 2B), while it was more than 95% without the point estimate constraint (Figure 2A) for higher variabilities and sample sizes. For greater differences between the products, it limits the probability of concluding BE even for higher variabilities and, most importantly, the increase in sample size seems to reduce the probability of showing BE (Figure 2D,F,H). This effect is somehow expected because when the differences increase, the point estimate constraint will progressively be predominant in the overall combined regulatory criterion, as previously demonstrated [19].

A T1E increase associated with the BE acceptance limits that are scaled in relation to the size of the within-subject variability have been expected since the very early presentation of these methods [20]. This came from the fact that the TOST (two one-sided test) procedure cannot be directly applied to the proposed method since the BE limits themselves become random variables and the method is not correct in the strict sense [3]. This inflation was later seen to be influenced by many factors, such as the regulatory constant value, the cut-off point between the unscaled and scaled ABE, as well as the existence of continuity at the cut-off point. Depending on the balance between these factors, T1E inflations of up to 16% have been described [13,19]. In the present case, as shown in Figure 3A, a T1E up to a maximum of 7% around a WSCV of 13.93% for sample sizes of more than 40 subjects is seen. This is in the same order of magnitude as already described for the EMA widening approach for HVDP [14]. It can also be seen that the surface of T1E inflation spreads from 12% to 27% of a WSCV, in a relatively independent manner for sample sizes higher than 40 subjects. For sample sizes below 40, this T1E inflation is not seen, maybe because for sample sizes of this order the demonstration of BE is always very difficult, and the power obtained should be intrinsically low. In most of the inflation area, T1Es were less than 6%. The inclusion of the point estimate constraint did not reduce the maximum T1E inflation around the WSCV of 13.93% (Figure 3B). This is similar to the conclusions reached by Endrenyi et al. [19], where no effect of the point estimate constraint was observed on consumer risk in the vicinity of the cut-off point. However, the inclusion of this constraint significantly reduces the area of T1E inflation to be contained between a WSCV of 12% to 18%, especially for higher sample sizes. The inflation of the T1E has obvious consequences in the quality of the employed statistical methods and several approaches have been put forward in order to solve this issue [14,21]. However, as for the EMA criteria for HVDP, it is fair to say that in practice the consumer risk is nearly 5% and since the proposed regulatory conditions are continuous around a WSCV of 13.93% the probabilities of acceptance and rejection are only slightly different on the two sides of the cut-off WSCV [19].

We have previously shown that the proposed NLIVR criterion, when compared to the current EMA NTI acceptance criterion, greatly reduces the sample sizes required to demonstrate BE for a WSCV above 15% when the two products are equal [9]. This sample size reduction is also observed, as expected, when small acceptable differences (from 2.5% to 7.5%) in the GMR are considered, a possible initial conditions considered by applicant’s when determining required sample sizes for BE clinical trials. Figure 4 shows that the sample sizes required for 80% power in a BE study for a WSCV above 15% is, again, greatly reduced when comparing the NLVIR to the current NTI EMA criterion in all the considered scenarios. This is, in fact, one of the major advantages expected for this type of “fixed multiple-of-CV” methods [20]. In addition to this, the simulations also show that the inclusion of the GMR constraint does not significantly change the sample size requirement, if the expected differences are low. Figure 4 and Table 1 show that if the differences are lower than 5%, the sample size required (for both 80% or 90% power) is basically the same either with or without the GMR constraint. This picture, however, changes dramatically if the expected difference between products increases above 5%. For the simulated difference of 7.5% and a WSCV above 20%, the inclusion of the GMR constraint greatly increases the sample size required to have at least 80% power. This is understandable since at large variabilities the probability of observing very high (or very low) GMR is higher, as already described [3]. In addition, the inclusion of this constraint should also work as an additional reason for not artificially increasing the clinical assay variability in order to conclude BE because, as can be seen in Figure 2, it reduces the power to conclude BE in a non-linear way in those cases.

It is acknowledged that this proposed method does not include a comparison of the intra-subject CV of the test and reference products, since this has never been required in the European Union. For a change in this paradigm, evidence that products may exhibit a >2-fold difference in the intra-subject variability would be essential. It seems rather unlikely, except in exceptional circumstances with notable differences in manufacturing technology, because intra-subject variability is mostly due to the bioanalytical method, especially if the CV is low, and the physiological processes involved in absorption and the first-pass effect that are affecting both products similarly. Therefore, imposing a comparison for intra-subject CV within a 2-fold acceptance limit was considered unnecessary and an increased burden for something that is not considered as a clinical concern presently [22]. Furthermore, large differences in manufacturing technology are not expected between generics and the innovator product, since generics tend to copy as much as possible the reference products to have greater success in ABE (e.g., solid dispersions for tacrolimus and everolimus, but simple manufacturing for BCS class I drugs like warfarin). In addition, Tothfalusi and Endrenyi further noted that “the additional regulatory criterion modifies the statistical features of the primary criterion that of comparing the means” [22].

## 5. Conclusions

The proposed average bioequivalence with narrowed limits based on the within-subject variability of the reference product (NLIVR) criterion for the evaluation of the bioequivalence of NTI drugs in the European Union clearly reduces the regulatory burden by lowering the sample size required in bioequivalence studies for drugs with moderate or high intra-subject CV at the expense of a replicate design. Since in Europe, the decision for considering a drug as NTI is made case-by-case and based on clinical considerations, this proposal may provide a balance between a stringent criterion when needed (for low WSCV) and without too many limitations when not needed (for medium-to-high WSCV). This may allow for considering more frequently the inclusion of Cmax in the NTI criteria and the inclusion of further molecules in the list of NTI drugs, both in an effort for global harmonization.

As in the case of the current HVDP EMA protocol, its statistical use results in an increase in the T1E, mainly in the vicinity of the cut-off point of a 13.9% WSCV. Besides this, in its original form, the probability of showing bioequivalence of products differing more than 10% is significant if the reference within-subject variability is also moderate-to-high. This, by itself should not be problematic. However, the inclusion of an additional GMR constraint, requiring that the point estimate of the GMR to be contained in the acceptance range of 90.00–111.11%, further limits this risk. The inclusion of this constraint has no major consequences in the regulatory burden if the product’s GMR differ less than 5% and, consequently, this GMR constraint should be included as an additional condition for the sake of not negatively affecting the public awareness on the quality and interchangeability of generic NTI medicines, that are still frequently titrated within each individual patient and their safety/efficacy controlled by therapeutic drug monitoring based on either PK or PD endpoints.

## Figures and Tables

**Table 1:** Sample sizes with the proposed NLIVR conditions with and without considering the GMR constraint, with different theoretical GMRs, reference with-subject variabilities and for a power of 80% and 90%.

				NLIVR			NLIVR + GMR Constraint			
	CV	GMR	1.000	0.975	0.950	0.925	1.000	0.975	0.950	0.925
80% Power	5%		6	6	9	33	6	6	9	33
	10%		12	15	30	111	12	15	30	111
	15%		24	30	57	195	24	30	57	195
	20%		27	27	42	78	27	27	42	81
	25%		27	27	33	54	27	27	36	78
	30%		27	27	33	45	27	27	36	111
	35%		33	33	39	51	33	33	42	150
	40%		39	42	48	66	39	42	57	192
90% Power	5%		6	6	12	45	6	6	12	45
	10%		15	21	42	153	15	21	42	153
	15%		30	39	78	273	30	39	78	273
	20%		30	36	57	108	30	36	57	129
	25%		30	33	48	72	33	36	51	180
	30%		33	36	45	60	36	39	69	258
	35%		39	42	51	69	42	48	90	348
	40%		48	51	66	87	54	63	117	450

CV: Coefficient of variation; GMR: Ratio of geometric means.

## Data Availability

Not Applicable.
